# A Deformable Motor Driven by Dielectric Elastomer Actuators and Flexible Mechanisms

**DOI:** 10.3389/frobt.2019.00001

**Published:** 2019-02-08

**Authors:** Ayato Minaminosono, Hiroki Shigemune, Yuto Okuno, Tsubasa Katsumata, Naoki Hosoya, Shingo Maeda

**Affiliations:** ^1^Department of Mechanical Engineering, Shibaura Institute of Technology, Tokyo, Japan; ^2^Department of Applied Physics, Waseda University, Tokyo, Japan

**Keywords:** dielectric elastomer actuator, internal stress, rotational performance, symmetry, strain, deformable

## Abstract

Soft robots with dynamic motion could be used in a variety of applications involving the handling of fragile materials. Rotational motors are often used as actuators to provide functions for robots (e.g., vibration, locomotion, and suction). To broaden the applications of soft robots, it will be necessary to develop a rotational motor that does not prevent robots from undergoing deformation. In this study, we developed a deformable motor based on dielectric elastomer actuators (DEAs) that is lightweight, consumes little energy, and does not generate a magnetic field. We tested the new motor in two experiments. First, we showed that internal stress changes in the DEAs were transmitted to the mechanism that rotates the motor. Second, we demonstrated that the deformable motor rotated even when it was deformed by an external force. In particular, the rotational performance did not decrease when an external force was applied to deform the motor into an elliptical shape. Our motor opens the door to applications of rotational motion to soft robots.

## Introduction

Traditional robots are generally made of hard materials, but soft robots, which are made of rubber, gels, or paper, can provide dynamic motion, and innate safety based on the properties of their constituent materials (Maeda et al., 2015, 2016; Hosoya et al., 2016a; Shigemune et al., 2016, 2017). The advantages of soft robots will promote the development of human–robot coexistence. For example, in contrast to hard robots, soft robots can handle many kinds of materials: hard or soft, fragile or robust, and thick or thin (Suzumori et al., 1992; Shintake et al., 2015b; Galloway et al., 2016; Okuno et al., 2018), which is difficult for hard robots.

Rotational motion has been applied in robotics (Anderson et al., 2010). In traditional robots, magnetic motors are normally used to produce rotational motion. In previous works, researchers have developed motors driven by soft actuators (Kornbluh et al., 1998; Anderson et al., 2010, 2011; O'Brien et al., 2010; Hwang and Higuchi, 2014; Ainla et al., 2017). Ainla et al. developed a motor based on a pneumatic actuator that functions by feeding air into a flexible structure (Diesel and Brock, 2013; Cacucciolo et al., 2016). Although the pneumatic actuator has the potential to produce a large force, an additional pump is needed to feed the air, causing the whole system required to drive the motor via the pneumatic actuator to be large and heavy. In other studies, Hwang et al. developed a rotational motor based on shape memory alloys (SMAs). SMAs can recover their shape by increasing their temperature (Minetaa et al., 2002). SMAs are capable of generating large strokes and forces. However, SMAs consume a lot of energy and have slow response times. Anderson et al. developed a rotational motor based on dielectric elastomer actuators (DEAs) (Anderson et al., 2010). DEAs consist of elastomers sandwiched between compliant electrodes. Applying a voltage to the electrodes generates electrostatic forces, which produces a large displacement (Pelrine et al., 2000; Madden et al., 2004; Hosoya et al., 2015). The advantages of DEAs include: (1) simple and lightweight structures, (2) compatibility with low energy consumption, (3) generation of large actuation strokes, and (4) fast response time (Plante and Dubowsky, 2007). In addition, DEAs can be driven under deformation. Anderson et al. proposed a mechanism for converting the expansion of DEA into rotational motion by building a crank mechanism. They showed that the performance (torque per weight) in a motor based on DEAs was higher than that of the traditional magnetic motors. Although the idea of DEA motors is unique, their motion and behavior are same as those of solid motors. Motors that can deform and operate in a deformed state of them would enable the development of novel soft machines that could function in a wide range of environments.

**Figure 1:**
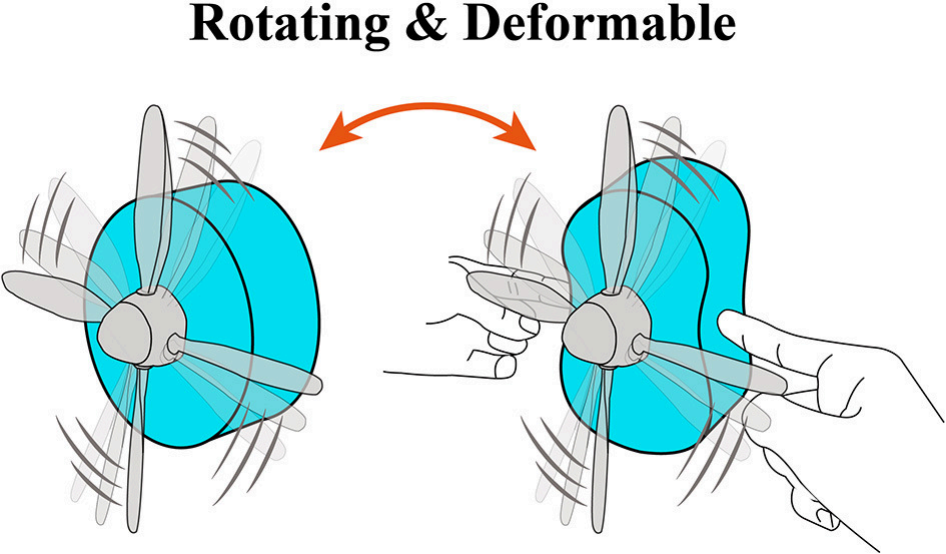
Concept of a deformable motor. The motor consists of flexible materials and deformable mechanisms.

Herein, we propose a deformable motor based on DEAs. Due to the flexibility of the frame and DEA, our deformable motor could rotate even when it was deformed by external force (Figure 1, Video S1). To create the new motor, we employed the mechanism proposed by Anderson et al. together with a flexible frame (Kofod et al., 2006; Shintake et al., 2015b). Furthermore, we succeeded in visualizing the dynamic stress changes of the deformable motor, and then used a high-speed polarization-imaging camera to obtain proof that the stress changes of the DEA led to rotational motion. Moreover, we revealed the relationship between rotational performance and the strain of the deformable motor.

## Mechanism

### DEA Activation

**Figure 2:**
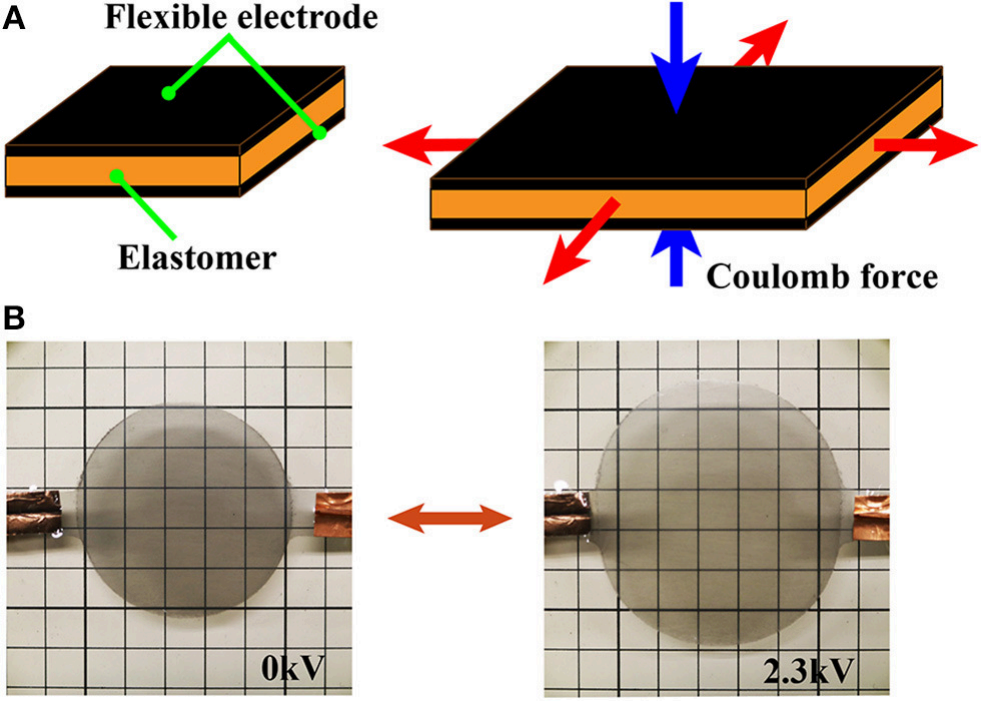
Three-dimensional models and images of the mechanisms in the deformable motor. **(A)** Pressure determined by Equation (1). **(B)** Activation of a DEA when a voltage is applied.

Figure 2A shows the structure of the DEA, which consists of flexible electrodes that sandwich a dielectric elastomer. The flexible electrodes are composed of conductive materials such as carbon powder, carbon grease, rubber, or hydrogels. Upon application of a voltage, electric charges accumulate on the stretchable electrodes and generate a Coulomb force between them. The Coulomb force compresses the dielectric elastomer, and the elastomer stretches in a perpendicular direction due to the incompressibility of the elastomer. Due to its elasticity, the elastomer returns to its original shape when the electric charges are removed from the electrodes. Equation 1 describes equivalent electrostatic Maxwell stress *P* (N/m^2^) induced by the compression force (Wissler and Mazza, 2007):

(1) $$P\;=\varepsilon_{r}\varepsilon_{0}E^{2}=\varepsilon_{r}\varepsilon_{0}{\left(\frac{V}{z}\right)}^{2},$$

where ε_*r*_ is the relative dielectric constant of the elastomer, ε_0_ is the permittivity of free space (ε_0_ = 8.854 × 10^−12^ F/m), *E* is the electric field (V/m), *V* is applied voltage (V), and *z* is the thickness of the elastomer (m). Figure 2B and Video S3 illustrates the operation of a DEA.

### Rotation and Deformation of the Motor

**Figure 3:**
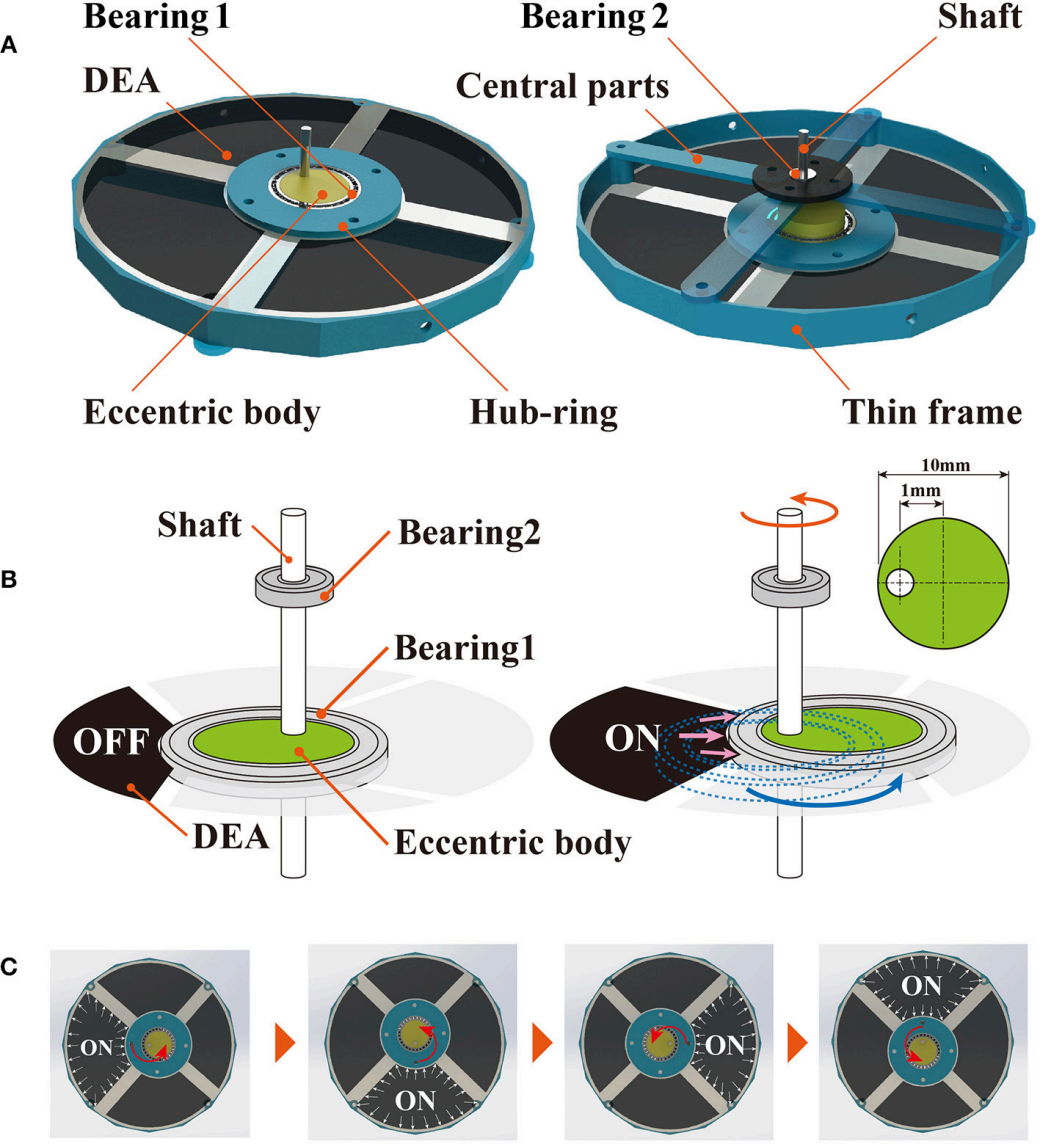
Mechanisms of the deformable motor. **(A)** Crank mechanism consists of bearing 1, bearing 2, eccentric body, and shaft. DEAs are fixed by a thin frame and hub-ring. **(B)** Rotational mechanism of the deformable motor. The crank moves under the force generated by the DEA. **(C)** The crank mechanism is activated when the four DEAs are stretched in order.

Figure 3A depicts the design for the deformable motor, which is composed of the thin frame, central parts, a crank mechanism, and four DEAs. We employed a thin frame to achieve deformation by application of an external force. The central parts have four joints and are connected to the four fulcrums of the frame in such a manner that the shaft is positioned at the center of the motor.

We classified previous motors for use with soft materials into two classes, based on the type of activation in the shaft: those in which the shaft of the motor moves around in a circle (O'Brien et al., 2010; Anderson et al., 2011), and those in which the shaft spins in the upright state (Kornbluh et al., 1998; Anderson et al., 2010). We employed the latter type of actuation; the shaft of our deformable motor spins in the upright state. Anderson et al. described a method for rotating the shaft by combining the crank mechanism with a DEA (Anderson et al., 2010). We used their proposed mechanism to convert the expansion of the DEA into rotational motion.

Here, we explain the mechanism underlying rotation of the deformable motor. The crank mechanism is composed of bearing 1, bearing 2, the eccentric body, and the shaft. When one of the four DEAs is expanded, the eccentric body moves around the shaft as shown in Figure 3B. Because the eccentric body and the shaft are integrated, the movement of the eccentric body rotates the shaft. We can then generate rotational motion by activating the four DEAs in order (Figure 3C, Video S6). Bearing 1 keeps the shaft at the center of the motor by connecting with the thin frame through the central parts shown in Figure 3A. By changing the eccentricity of the eccentric body, we can adjust the maximum torque and rotational speed of the motor.

**Figure 4:**
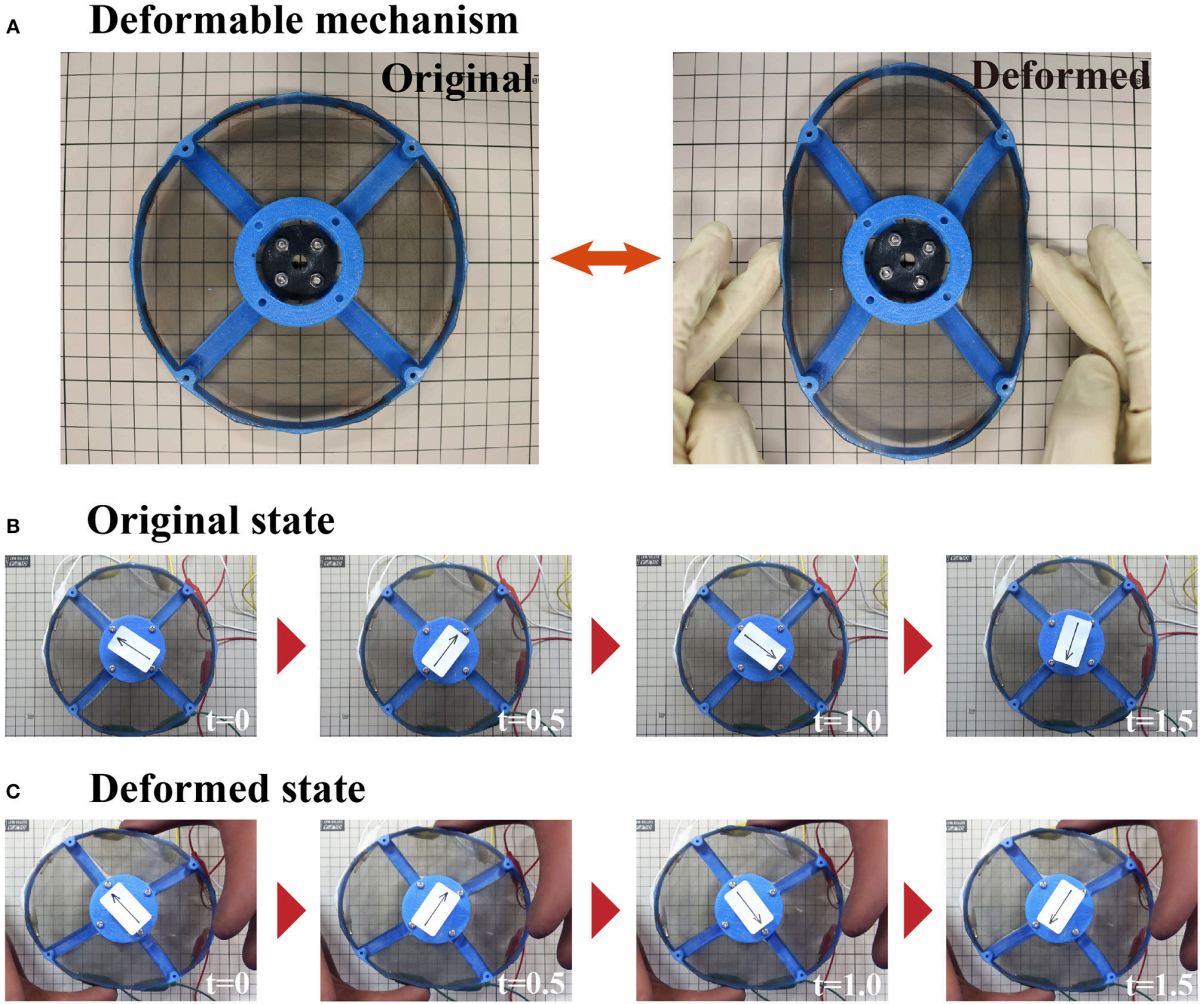
The deformable mechanism and images of the motor under deformation. **(A)** The deformable mechanism consists of the thin frame and central parts. **(B)** Time-series imagines of a rotating deformable motor. The motor rotates without deforming the frame. **(C)** Motor rotating within a deformed frame.

Figure 4 shows the activation of the deformable motor. When the frame deforms as shown in Figure 4A, eight joints work to arrange the shaft at the center of the motor. Figure 4B shows the rotational motion without the external force (Original state). Figure 4C shows the rotational motion of the motor when an external force is applied from the top and bottom sides (Deformed state). In the figure, an arrow has been placed at the center of the motor to clarify the direction of rotation. Notably, the motor works while under deformation.

## Fabrication

### Deformable Motor

To prepare DEAs, we used VHB Y-4905J (VHB Y-4905J; 3M, Maplewood, MN, USA) as the elastomer and multi-walled carbon nanotubes (MWNTs) (multi-walled carbon nanotube 724769; Sigma-Aldrich, Saint Louis, MO, USA) as the electrode. According to Equation 1, the relative dielectric constant of the elastomer affects the extension of the DEA. The dielectric constant of VHB Y-4905J is 4.8, and the strain of a DEA made with VHB Y-4905J is >300% (Anderson et al., 2010). In addition, the extension of a DEA made with 300% pre-strained VHB is larger than that of a silicon DEA (Pelrine et al., 2000). We applied 300% pre-strain to the elastomer because pre-straining makes the elastomer softer (Pelrine et al., 2000), and employed MWNTs as electrodes because DEAs made with MWNTs have high work density (Hughes and Spinks, 2005). The MWNTs were applied to the elastomer by brushing (Shigemune et al., 2018). To reduce friction and weight, for the crank mechanism we selected an eccentric body, bearings, and a rotating shaft that were of small scale. We employed an eccentric body with a diameter of 10 mm and a 1-mm gap from center of the circle, as shown in Figure 3B. The diameters of the rotating shaft and the inner diameter of the Bearing 2 were both 3 mm. We used a three-dimensional printer (Dreamer; Flashforge, Jinhua, China) and an ABS resin (ABS 600 g; FlashForge) to fabricate the frame of the motor and the parts for placement of the rotational shaft at the center. Because ABS resin is ductile, the frame is not likely to be broken by deformation (Perez et al., 2014).

### Controller

**Figure 5:**
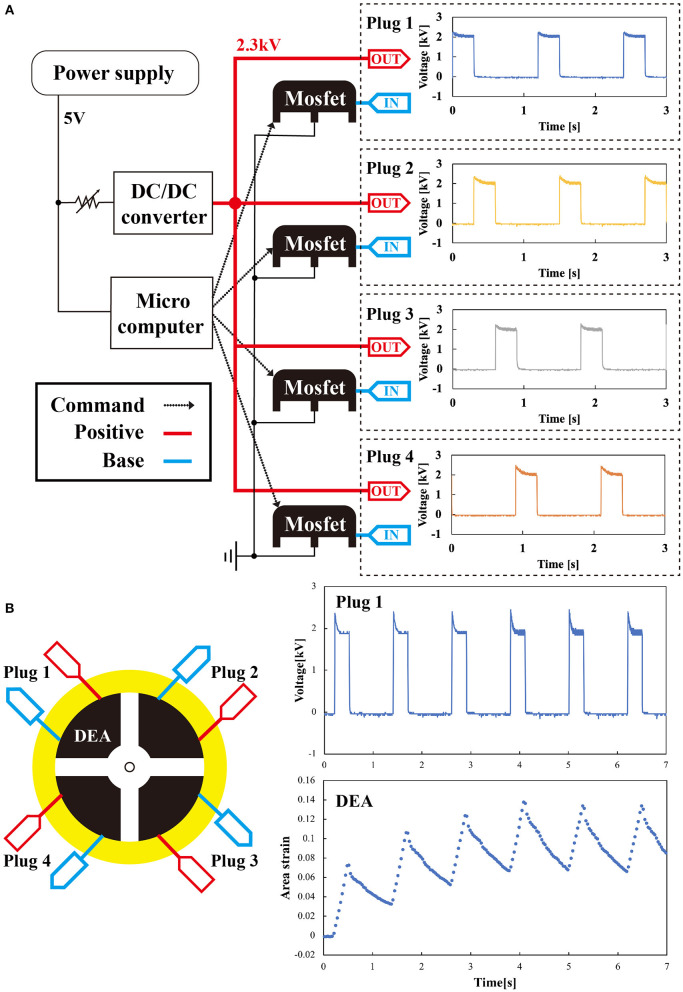
System for connecting the controller to the deformable motor **(A)** system diagram of the controller and the waveform of the voltage outputted from the Plugs 1–4. **(B)** Signal of the controller and the response of the DEA to the signal. As time passes, the area strain in one cycle gradually stabilizes.

To drive the deformable motor, we developed a circuit to control the four DEAs. DEAs have the advantage of high energy efficiency per unit weight. When the circuit that controls the DEAs becomes large, the system loses this efficiency. Hence, we developed a compact control circuit to maintain the energy efficiency advantage. We designed a controller that drove the four DEAs with one DC/DC converter. Figure 5 shows the system of the controller and how the controller is connected to the deformable motor. The controller consists of a microcomputer (Nucleo-f 401 re), a DC/DC converter (EMCO Q 101-5), and power MOSFETs (IXTH 02 N 450 HV). The microcomputer outputs signals to each DEA (plugs 1–4). The DC/DC converter is required to generate the high voltage that drives the DEA. Previous studies demonstrated that EMCO Q 101-5 works acceptably on robots made with DEAs (Wingert et al., 2006; Ahmadi et al., 2012; Shintake et al., 2015a). We set the maximum output to 3 kV because electrical breakdown within the DEAs occurred above this voltage. The power MOSFETs can switch the voltage up to 4.5 kV. We used the same power supply to provide energy to both the microcomputer and the DC/DC converter.

We measured the output voltage produced from the controller. IN and OUT of the controller in Figure 5A were connected to each DEA in the motor shown in Figure 5B. Physically, the controller and the DEAs were connected by a copper tape. To provide high voltage, we clamped the copper tape with clips from the controller. The right of Figure 5A shows waveforms of the voltage outputted from the four plugs (Plugs 1–4). To measure the voltage, we directly connected plugs of the controller to an oscilloscope. We used the following conditions: output voltage of 2 kV, frequency of 0.833 Hz, and a duty ratio with a rectangular pulse wave of 25%. The waveforms showed transient characteristics, which are attributed to the thermal resistance of the power MOSFET. Each of the four outputs converged to 2 kV, and we confirmed that the controller could output and control the voltage to drive the DEA. The right panel of Figure 5B shows that the behavior of the DEA reached a steady state after 4 s. The maximum area strains of the DEAs controlled by the system were ~114%.

## Experiments

### Visualization of the Internal Stress Distribution

**Figure 6:**
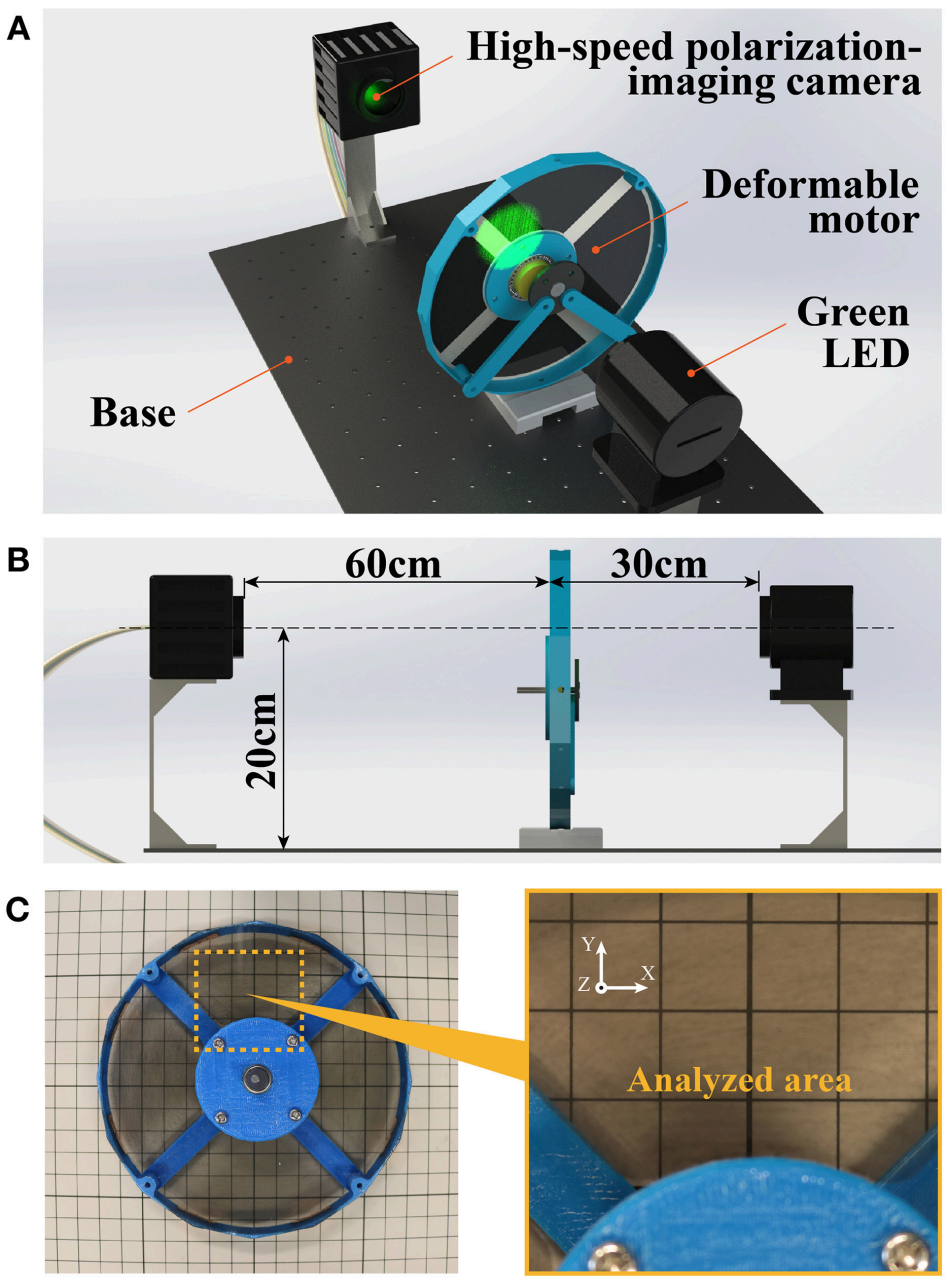
Experimental setup for measurement of internal stress. **(A)** Green light penetrates the deformable motor, and birefringence information is transmitted to the camera. **(B)** Placement of the experimental instrument. The light and lens of the camera are in a straight line. **(C)** Analysis area is 40 × 40 mm.

To determine whether the deformable motor could be driven by the DEAs, we visualized the spatio-temporal changes of stress distribution inside the DEA while driving the deformable motor. Previous studies showed that the motors rotate under force from the DEAs, and proved that the mechanism works in a simulated environment. However, those reports never confirmed the application of the force to the motor in an actual environment. Figure 6 shows the experimental setup used to visualize the internal stress distribution in the DEA. For this experiment, we used a high-speed polarization-imaging camera and an LED light (Figure 6A). This camera is capable of measuring the birefringence phase difference (birefringence) of transparent materials (Hosoya et al., 2016b,c, 2017a,b).

We used the birefringence of DEA as a parameter to indicate the condition of a material's stress and structure. During measurement of a thin film with a uniform thickness, the material's birefringence changes due to internal stress, such as the pre-strain and coulomb force of the DEA. Therefore, birefringence is correlated with relative stress. For example, phase difference is large in areas with large stress.

The experimental setup was as follows: three camera and motor were 60 cm apart, whereas the motor and the light were 30 cm apart (Figure 6B). The center of the measurement area was 20 cm above the fixed base. The high-speed polarization-imaging camera had a speed of 125 fps, an exposure time of 5 s, and a visualization area of 40 × 40 mm. Figure 6C shows the visualization area. The light source was a green LED (operating wavelength: 480–540 nm; bandwidth of band-pass filter: 520 ± 10 nm; power of incident light: 2.5 W/m^2^). The driving voltage of the DEA was 2.3 kV, the driving frequency was 15 Hz with a rectangular wave, and the duty ratio was 25%. The phase difference of the driving frequency among the four DEAs was π/2, and the four DEAs functioned in order.

**Figure 7:**
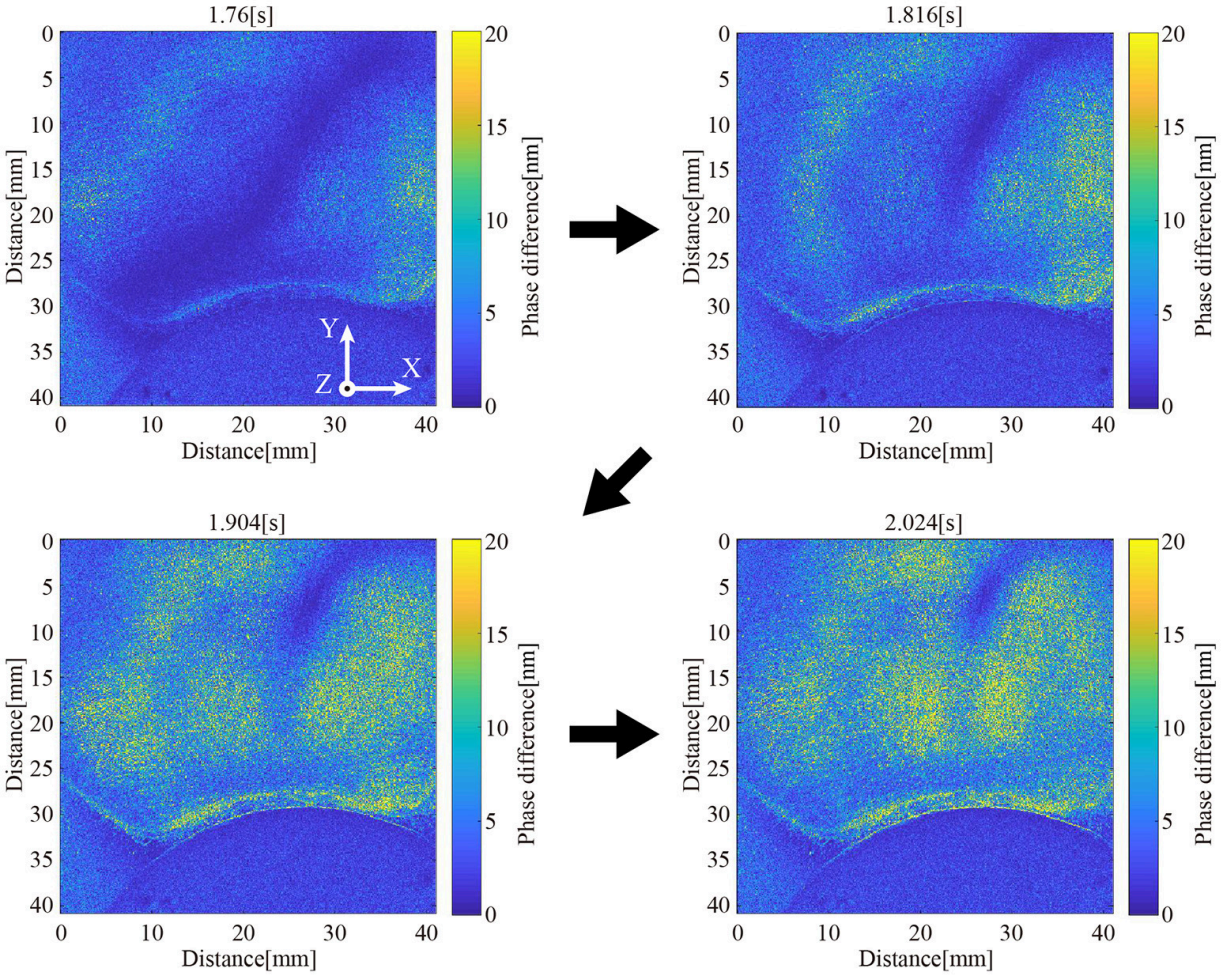
Visualization of internal stress distribution. The internal stress distribution changes upon application of a voltage to the DEA.

Figure 7 visualizes the spatio-temporal change of the stress inside the DEA via the high-speed polarization-imaging camera. The stress inside the DEA increased with the driving voltage. In addition, high stress occurred at the center part and the boundary between the DEA and the hub-ring. The stress at the center was a compressive stress in the vertical direction of the DEA (z-axis direction in Figure 7). We assume that this stress affects the rotational motion of the deformable motor. On the other hand, we presume that the stress at the boundary between the DEA and hub-ring compressed in the negative direction of the Y axis, as shown in the visualized phase of 2.024 s (Figure 7).

Furthermore, we could determine the rotational performance of the deformable motor from this experiment. We confirmed high internal stress in almost all of the area after ~0.25 s from activation of the DEA. In other words, the motor required a minimum of 0.25 s to move the crank by the DEA when 2.3 kV was applied. It takes 1 s to rotate the motor with the four DEAs. We then presumed that the deformable motor rotates at 60 rpm under the fastest condition. Eventually, we designed a control program for the deformable motor with a maximum speed of 60 rpm.

### Mechanical Characteristics of the Deformable Motor

#### Setup

**Figure 8:**
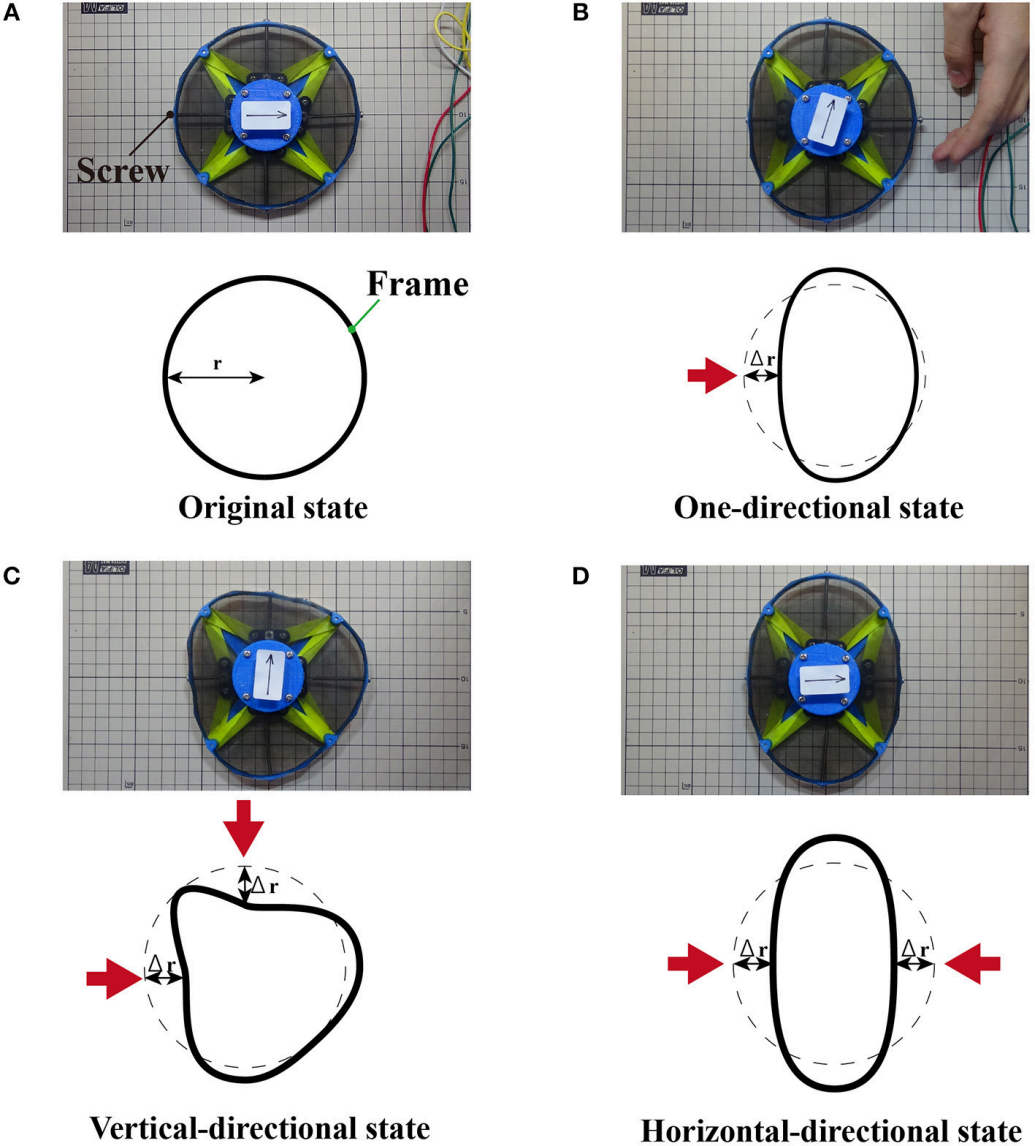
Experimental setup. **(A)** Original state without deformation. **(B)** The one-directional state deforms the motor from one direction. **(C)** The vertical-directional state deforms the motor from one horizontal and one vertical direction. **(D)** The horizontal-directional state deforms the motor from two horizontal directions.

To elucidate the performance of the deformable motor, we measured its mechanical characteristics. We designed the motor to be capable of deformation into various shapes. Here, we defined three deformed states to compare the mechanical characteristics of each state. To quantify the deformation, we added four sets of long screws, vertical spacers, and parts with two joints. Figure 8 shows each state deformed by the additional parts. Turning a screw clockwise places pressure on one point of the frame, and the radius *r* [mm] from the shaft changes as shown in Figure 8A. The pitch of the screw was 0.5 mm. Thus, one turn of the screw decreased the radius by 0.5 mm. Figure 8A and Video S4 illustrate the original state. Figure 8B and Video S5 show a one-directional state. Figures 8C and D show a vertical-directional state, and a horizontal-directional state respectively. Equation (2) derives the radial strain δ of the frame:

(2) $$\delta =\frac{\Delta r}{r}=\frac{0.5}{r}\left(n+\frac{\theta }{360}\right),$$

where *r*[mm] is the radial displacement, *n* is the number of screw rotations, and θ [°] is the rotation angle, which is added to the rotational times.

We investigated the relationship between the radial strain and the maximum torque. Additionally, we examined the radial strain and the maximum rotational speed when the motor was deformed. We set the two radial strains of the horizontal-directional and vertical-directional states to the same value in order to define the type of the deformed states.

**Figure 9:**
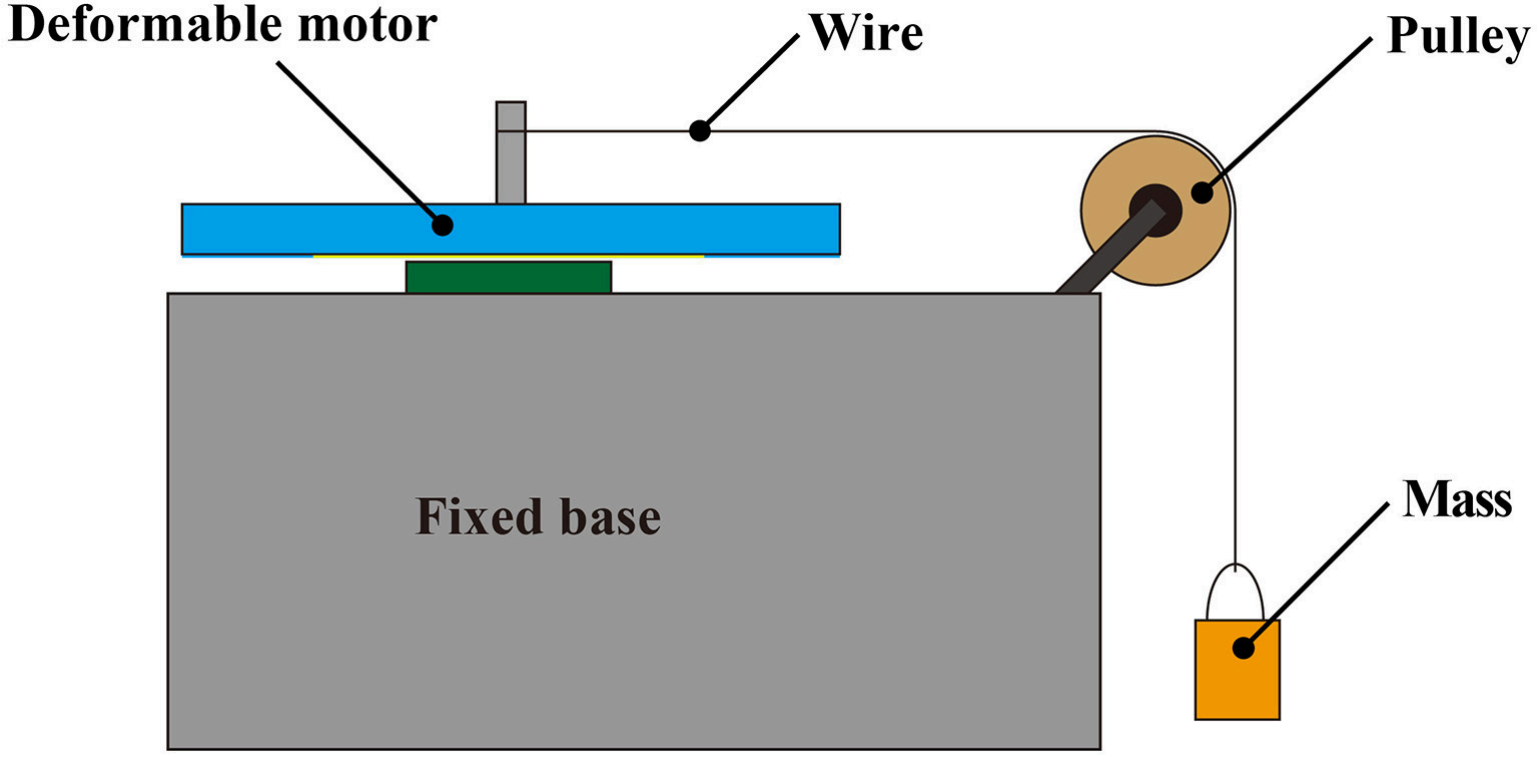
Experimental device for measuring torque. To reduce interference from gravity, the shaft of the motor is placed in a vertical orientation relative to the ground.

Figure 9 illustrates an experimental setup used to measure the torque of the deformable motors. The motor shaft was placed in the vertical orientation relative to the ground, and the wire was placed parallel to the ground. We suspended a weight from the tip of the wire to apply torque on the shaft. The length *L* [cm] of the wire from the pulley to the mass was 30 cm. The weight of the mass included the weight of the wire.

Equation (3) derives the torque *T* [mN·m] of the deformable motor:

(3) $$T=rF=r\left(w_{i}+w_{o}\right)g;\;xw_{i}=kL,$$

where *r* [m] is the radius of the shaft, *F* [mN] is the external force, *w*_*i*_ [*g*] is the weight of the wire, *w*_*o*_ [*g*] is the weight of the mass, *g* is the gravitational acceleration (*g* = 9.81 m/s^2^), and *k* is the weight per wire length (*k* = 0.0024 g/cm ). We neglected the friction of the pulley.

#### Results and Discussion

**Figure 10:**
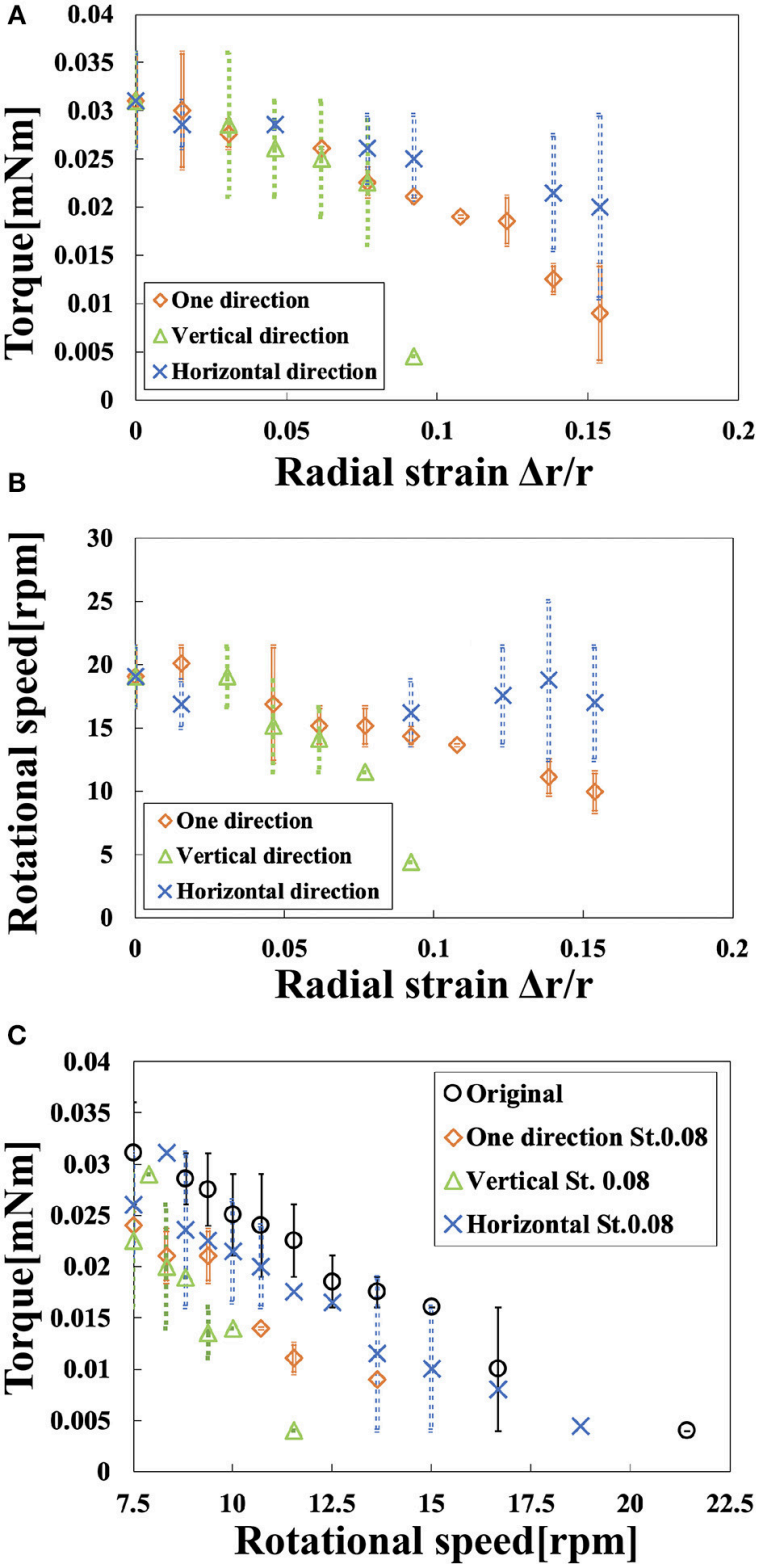
Rotational performance. **(A)** Torque vs. radial strain in the three deformed states. **(B)** Rotational speed vs. radial strain in the three deformed states. **(C)** Motor torque vs. rotational speed.

Figure 10A shows the relationship between the radial strain and the torque in the three deformed states. Table 1 shows the torque generated by the motor at each deformed state when the radial strain was 0, 0.09, and 0.15. We conducted the experiment twice; Table 1 shows the average, maximum, and the minimum values of the torque in the two replicates. The torque of the original state was 0.031 mN m. The one-directional state had a torque of 0.009 mN m when the radial strain was 0.15. The vertical-directional state had a torque of 0.004 mN m when the radial strain was 0.09. When the radial strain was over 0.1 in the vertical-directional state, the motor could not rotate. The torque of the horizontal-directional state was 0.021 mN m when the radial strain was 0.15. As shown in Figure 10A, the torque of the horizontal-directional state did not change significantly when a large strain was applied. We observed a significant decrease in the torque in the vertical-directional state. Thus, the nature of the deformed state affects the torque of the motor.

**Table 1:** Torque experiment.

	**Strain 0 [mN m] (Original state)**			**Strain 0.09 [mN m]**			**Strain 0.15 [mN m]**		
	**AVE**	**MAX**	**MIN**	**AVE**	**MAX**	**MIN**	**AVE**	**MAX**	**MIN**
One-directional	0.031	0.036	0.026	0.021	0.021	0.021	0.009	0.014	0.004
Vertical				0.004	0.009	0.000	–	–	–
Horizontal				0.026	0.029	0.021	0.021	0.026	0.014

Figure 10B shows the relationship between radial strain and the rotational speed in the three deformed states. Table 2 shows the rotational speed generated by the motor in each deformed state when the radial strain was 0, 0.09, and 0.15. We also conducted this experiment twice; Table 2 includes the average, maximum, and minimum values of the rotational speed in the two attempts. The rotational speed of the original state was 19 rpm. When the radial strain was 0.15, the one-directional state had the rotational speed of 9.93 rpm, half the speed of the original state. The vertical-directional state had a torque of 4.4 rpm when the radial strain was 0.09. The rotational speed of the horizontal-directional state was 16.2 rpm when the radial strain was 0.15. As shown in Figure 10B, the rotational speed did not change significantly when a large strain was applied in the horizontal-directional state. In the three deformed states with a strain of 0.09, the vertical-directional state exhibited the worst performance, as in the torque experiment. Thus, rotational speed behaved similarly to torque.

**Table 2:** Rotational speed experiment.

	**Strain 0 [rpm] (Original state)**			**Strain 0.09 [rpm]**			**Strain 0.15 [rpm]**		
	**AVE**	**MAX**	**MIN**	**AVE**	**MAX**	**MIN**	**AVE**	**MAX**	**MIN**
One-directional	19.0	21.4	16.7	14.3	15.0	13.6	9.9	11.5	8.3
Vertical				4.4	8.8	0.0	–	–	–
Horizontal				16.2	18.8	13.6	17.0	21.4	12.5

Figure 10C shows the relationship between rotational speed and torque. At a strain of 0.08 rotational performance decreased in the following order: original, horizontal-directional, one-directional, and vertical-directional. In other words, we revealed that the elliptical shape of the motor prevented its rotational performance from dramatically decreasing in the three deformed states. This property, i.e., that torque decreased as rotational speed increased, matched the features of the magnetic motor.

**Figure 11:**
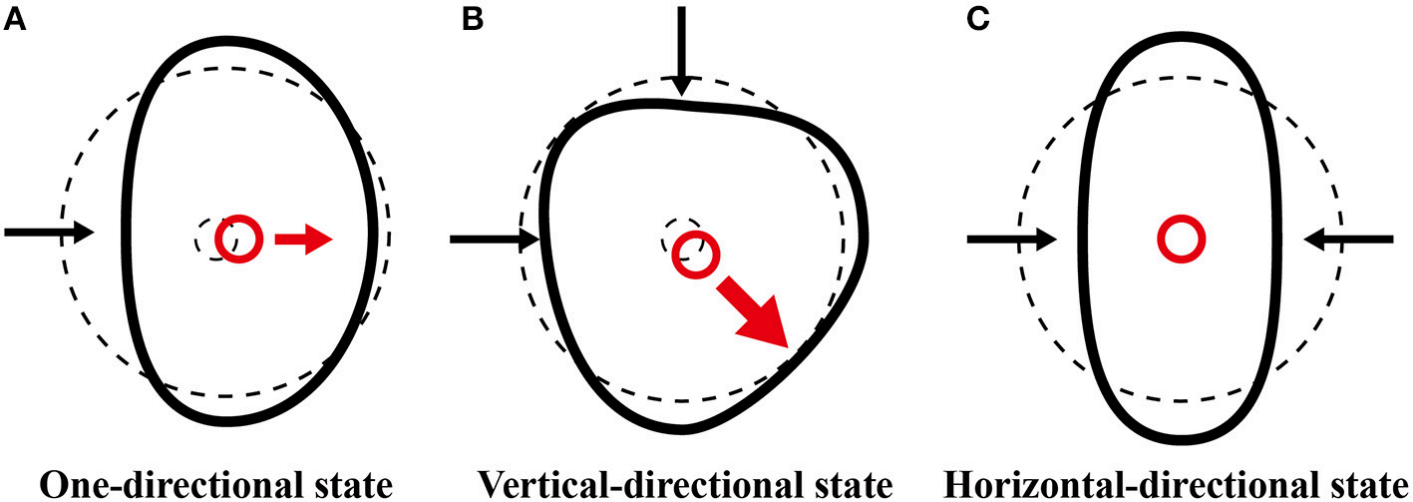
Diagram showing the force applied to the crank. **(A)** In the original state, the force applied to the crank is small. **(B)** In the vertical-directional state, the force applied to the crank is large. **(C)** In the horizontal-directional state, the forces applied to the crank cancel.

The performance of the deformable motor was strongly affected by the friction on the bearing in the crank mechanism. When the force acts on the crank, the rotational performance of the motor decreases because the friction on the bearing increases. The direction of the force applied to the crank represents the summation of the external force vectors. In the vertical-directional state, a large force acts on the crank in the lower right direction, as shown in Figure 11B. The force is smaller in the one-directional state than in the vertical-directional state, as shown in Figure 11A. On the other hand, the force does not act on the horizontal-directional state because the force vectors applied to the frame cancel, as shown in Figure 11C. Eventually, the friction of the bearing becomes the smallest in the horizontal-directional state. Hence, the horizontal-directional state demonstrates the best performance in the three deformed states. These results demonstrate that the symmetricity of the frame affects the rotational performance of the deformable motor.

Although the motor in the horizontal-directional state maintains the symmetricity, the rotational performance is lower than that of the original state. We conjectured that a decrement in the displacement of the DEAs degraded the performance of the motor. In the area of the frame that deforms toward the center of the motor, the pre-strain of the DEA close to the area becomes small. As the pre-strain of the DEA decreases, the elastic modulus of the DEA elastomer increases (Pelrine et al., 2000), and the displacement of the DEA decreases. This decrement in the displacement caused the performance of the motor to be lower in the horizontal-directional state than in the original state.

Eventually, because the effects of the friction of the crank or the displacement of the DEAs were small, the motor rotated in the all deformed states when the radial strain was <0.07. The motor worked acceptably in the horizontal-directional state because of the symmetrical deformation.

## Conclusion

We developed a deformable motor that can rotate its shaft under deformation due to the flexibility of its frame and DEAs. By visualizing the internal stress distribution, we observed the force generated by the DEA. The results revealed that the DEAs in the motor drove the crank mechanism to rotate the deformable motor. We also conducted an experiment to clarify the relationship between the rotational performance and deformations of the motor. When the deformation of the motor was small, the rotational speed and torque were almost same as those of the undeformed motor (strain, 0.07). Moreover, in the horizontal-directional state, a large deformation (strain 0.15) did not cause a significant decline in rotational speed or torque. A deformable motor with one layer of DEA yielded 2% of the torque of a traditional electromagnetic motor (RE-280RA; Mabuchi Motor, Chiba, Japan; deformable motor: 0.03 mN m; traditional electromagnetic motor: 1.47 mN m). We deduced that the performance of the deformable motor could be improved by stacking the DEAs. Theoretically, a motor using 48 layers of DEAs (thickness: 1.5 mm) would generate 48 times as much torque. We believe that stacking DEA layers has the potential to increase torque without making the motor enormous. In regard to energy efficiency per weight, our deformable motor generated 0.30 mN m/W g, whereas the traditional electromagnetic motor and one with DEAs (Anderson et al., 2010) generated 0.023 and 2.91 mN m/W g, respectively. The basic performances of the deformable motor can be improved by optimizing the design and materials (Video S2). In future studies, we hope to improve the performance of the deformable motor. Development of a deformable motor opens the possibility of applying rotational motion to soft robots.

## Author Contributions

All authors made intellectual contributions to this paper and approve of its publication. In particular, AM conducted the device design, experimental setup, data analysis, and participated in writing the manuscript. TK and NH designed the experimental setup and participated in writing the manuscript. HS and SM provided advice about the fundamentals of this research and participated in writing the manuscript.

### Conflict of Interest Statement

The authors declare that the research was conducted in the absence of any commercial or financial relationships that could be construed as a potential conflict of interest.

## Supplementary Material

Video S1 A deformable motor rotating with deformed state.

Video S2 A deformable motor rotating with centrifugal force.

Video S3 Activation of a dielectric elastomer actuator.

Video S4 Rotation of a deformable motor in an original state.

Video S5 Rotation of a deformable motor in an one-directional state.

Video S6 Rotational mechanism of a deformable motor.

## References

[B1] Ahmadi S. Mattos A. C. Barbazza A. Soleimani M. Boscariol P. Menon C. (2012).Fabrication and performance analysis of a DEA cuff designed for dry-suit applications. Smart Mater. Struct. 22:035002 10.1088/0964-1726/22/3/035002

[B2] Ainla A. Verma M. S. Yang D. Whitesides G. M. (2017). Soft, rotating pneumatic actuator. Soft Robot. 4, 297–304. 10.1089/soro.2017.0017 29182081

[B3] Anderson I. A. Hale T. Gisby T. Inamura T. McKay T. O'Brien B. (2010). A thin membrane artificial muscle rotary motor. Appl. Phys. A Mater. Sci. Process. 98, 75–83. 10.1007/s00339-009-5434-5

[B4] Anderson I. A. Tse T. C. H. Inamura T. O'Brien B. M. McKay T. Gisby T. (2011). A soft and dexterous motor. *Appl. Phys*. Lett. 98:123704 10.1063/1.3565195

[B5] Cacucciolo V. Renda F. Poccia E. Laschi C. Cianchetti M. (2016). Modelling the nonlinear response of fibre-reinforced bending fluidic actuators. Smart Mater. Struct. 25:105020 10.1088/0964-1726/25/10/105020

[B6] Diesel R. Brock O. (2013). A compliant hand based on a novel pneumatic actuator. robotics and automation (ICRA), in 2013 IEEE International Conference on Robotics and Automation (Karlsruhe), 2047–2053.

[B7] Galloway K. C. Becker K. P. Phillips B. Kirby J. Licht S. Tchernov D.. (2016). Soft robotic grippers for biological sampling on deep reefs. Soft Robot. 3, 23–33. 10.1089/soro.2015.0019 27625917 PMC4997628

[B8] Hosoya N. Baba S. Maeda S. (2015). Hemispherical breathing mode speaker using a dielectric elastomer actuator. J. Acoust. Soc. Am. 138, EL424–EL428. 10.1121/1.4934550 26520355

[B9] Hosoya N. Kajiwara I. Umenai K. (2016a). Dynamic characterizations of underwater structures using non-contact vibration test based on nanosecond laser ablation in water: investigation of cavitation bubbles by visualizing shockwaves using the Schlieren method. J. Vibrat. Control 22, 3649–3658. 10.1177/1077546314564693

[B10] Hosoya N. Kajiwara I. Umenai K. Maeda S. (2017b). Dynamic characterizations of underwater structures using noncontact vibration tests based on nanosecond laser ablation in water: evaluation of passive vibration suppression with damping materials. J. Vib. Control. 140, 486–492. 10.1177/1077546317710158

[B11] Hosoya N. Mishima M. Kajiwara I. Maeda S. (2017a). Non-destructive firmness assessment of apples using a non-contact laser excitation system based on a laser-induced plasma shock wave. Postharvest Biol. Technol. 128, 11–17. 10.1016/j.postharvbio.2017.01.014

[B12] Hosoya N. Terashima Y. Umenai K. Maeda S. (2016b). High spatial and temporal resolution measurement of mechanical properties in hydrogels by non-contact laser excitation. AIP Adv. 6:095223 10.1063/1.4964305

[B13] Hosoya N. Umino R. Kajiwara I. Maeda S. Onuma T. Mihara A. (2016c). Damage detection in transparent materials using non-contact laser excitation by nano-second laser ablation and high-speed polarization-imaging camera. Exp. Mech. 56, 339–343. 10.1007/s11340-015-0089-y

[B14] Hughes M. Spinks G. M. (2005). Multiwalled carbon-nanotube actuators. Adv. Mater. 17, 443–446. 10.1002/adma.200401076

[B15] Hwang D. Higuchi T. (2014). A rotary actuator using shape memory alloy (SAM) wires. IEEE/ASME Trans. Mech. 19, 1625–1635. 10.1109/TMECH.2013.2290545

[B16] Kofod G. Paajanen M. Bauer S. (2006). Self-organized minimum-energy structures for dielectric elastomer actuators. Appl. Phys. A 85, 141–143. 10.1007/s00339-006-3680-3

[B17] Kornbluh R. Pelrine R. Eckerle J. Joseph J. (1998). Electrostrictive polymer artificial muscle actuators, in IEEE International Conference on Robotics & Automation (Leuven), 2147–2154.

[B18] Madden J. D. W. Vandesteeg N. A. Anquetil P. A. Madden P. G. A. Takshi A. Pytel R. Z. (2004). Artificial muscle technology: physical principles and naval prospects. IEEE J. Ocean. Eng. 29, 706–728. 10.1109/JOE.2004.833135

[B19] Maeda S. Kato T. Kogure H. Hosoya N. (2015). Rapid response of thermo-sensitive hydrogels with porous structures. Appl. Phys. Lett. 106:171909 10.1063/1.4919585

[B20] Maeda S. Kato T. Otsuka Y. Hosoya N. Cianchetti M. Laschi C. (2016). Large deformation of self-oscillating polymer gel. Phys. Rev. E 93:010501. 10.1103/PhysRevE.93.010501 26871011

[B21] Minetaa T Mitsuia T. Watanabea Y. Kobayashia S. Hagab Y. Esashib M. (2002). An active guide wire with shape memory alloy bending actuator fabricated by room temperature process. Sens. Actuat. A 97–98, 632–637. 10.1016/S0924-4247(02)00021-3

[B22] O'Brien B. M. Calius E. P. Inamura T. Xie S. Q. Anderson I. A. (2010). Dielectric elastomer switches for smart artificial muscles. Appl. Phys. A. Mater. Sci. Process. 100, 385–389. 10.1007/s00339-010-5857-z

[B23] Okuno Y. Shigemune H. Kuwajima Y. Maeda S. (2018). Stretchable suction cup with electro adhesion. Adv. Mater. Technol. 4:1800304 10.1002/admt.201800304

[B24] Pelrine R. Kornbluh R. Pei Q. Joseph J. (2000). High-speed electrically actuated elastomers with strain greater than 100%. Science 287, 836–839. 10.1126/science.287.5454.836 10657293

[B25] Perez A. R. T. Roberson D. A. Wicker R. B. (2014). Fracture surface analysis of 3D-printed tensile specimens of novel ABS-based materials. J. Fail. Anal. Prevent. 14, 343–353. 10.1007/s11668-014-9803-9

[B26] Plante J. S. Dubowsky S. (2007). On the performance mechanisms of dielectric elastomer actuators. Sen. Actuat. A 137, 96–109. 10.1016/j.sna.2007.01.017

[B27] Shigemune H. Maeda S. Cacucciolo V. Iwata Y. Iwase E. Hashimoto S. (2017). Printed paer robot driven by electrostatic actuator. IEEE Robot. Autom. Lett. 2,1001–1007. 10.1109/LRA.2017.2658942

[B28] Shigemune H. Maeda S. Hara Y Hosoya N. Hashimoto S. (2016). Origami robot: a self-folding paper robot with an electrothermal actuator created by printing. IEEE/ASME Trans. Mech. 21, 2746–2754. 10.1109/TMECH.2016.2593912

[B29] Shigemune H. Sugano S. Nishitani J. Yamaguchi M. Hosoya N. Hashimoto S. (2018). Dielectric elastomer actuator with carbon nanotube electrodes painted with a soft brush. Actuators 7:51 10.3390/act7030051

[B30] Shintake J. Rosset S. Schubert B. Floreano D. Shea H. (2015b). Versatile soft grippers with intrinsic electroadhesion based on multifunctional polymer actuators. *Adv*. Mater. 28, 231–238. 10.1002/adma.201504264 26551665

[B31] Shintake J. Rosset S. Schubert B. E. Floreano D. Shea R. (2015a). A foldable antagonistic actuator. IEEE/ASME Trans. Mech. 20, 1997–2008. 10.1109/TMECH.2014.2359337

[B32] Suzumori K. Eikura S. Tanaka H. (1992). Applying a flexible microactuator to robotic mechanisms. IEEE Control Syst. 12, 21–27.

[B33] Wingert A. Lichter M. D. Dubowsky S. (2006). On the design of large degree-of-freedom digital mechatronic devices based on bistable dielectric elastomer actuators. IEEE/ASME Trans. Mech. 11, 448–456. 10.1109/TMECH.2006.878542

[B34] Wissler M. Mazza E. (2007). Electromechanical coupling in dielectric elastomer actuators. Sens. Actuat. A. 138, 384–393. 10.1016/j.sna.2007.05.029

